# The Mammary Artery Lifeline: How Collateral Dependency of Hidden Aortic Occlusion Altered Revascularization Strategy

**DOI:** 10.1016/j.jaccas.2025.106129

**Published:** 2025-12-03

**Authors:** Georgia R. Layton, Ajaay Shrinivas, Ibrahim Antoun, Hiwa Sherzad, Marios Patronis, Mustafa Zakkar

**Affiliations:** a Department of Cardiac Surgery, University Hospitals of Leicester NHS Trust, Leicester, United Kingdom; b Department of Cardiovascular Sciences, University of Leicester, Leicester, United Kingdom; c Department of Cardiology, University Hospitals of Leicester NHS Trust, Leicester, United Kingdom

**Keywords:** acute coronary syndrome, coronary angiography, coronary artery bypass, peripheral vascular disease

## Abstract

**Background:**

The mammary arteries are commonly used as an arterial conduit during coronary artery bypass grafting (CABG). However, in a small proportion of patients, unrecognized extracardiac vascular pathology may render internal mammary artery harvest catastrophic during CABG.

**Case Summary:**

A 61-year-old man presented with non–ST-segment elevation myocardial infarction and was referred for CABG. Preoperative computed tomography (CT) angiography revealed complete infrarenal aortic occlusion with extensive collateral perfusion of the pelvis and lower limbs, predominantly via the right internal mammary artery, as well as the intercostal, subcostal, and lumbar arteries. CABG was deemed prohibitively high risk owing to risk of catastrophic ischemia. The patient underwent staged percutaneous coronary intervention, with good recovery.

**Discussion:**

This case underscores the importance of CT imaging in high-risk CABG candidates at risk of peripheral arterial disease, such as those with absent femoral pulses or claudication. Recognition of critical collateral networks prevents inadvertent conduit harvest and life-threatening ischemia.

**Take-Home Messages:**

CT aortogram in selected candidates safeguards conduit selection and can avert catastrophic perioperative compromise. Coronary management should be individualized through multidisciplinary evaluation, as guideline-based strategies may not account for complex anatomical variations.

## Graphical Abstract

**Figure:**
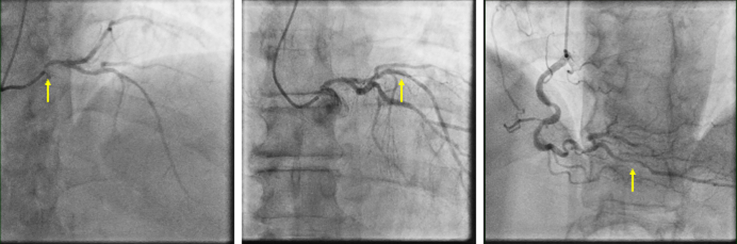

## History of Presentation

A 61-year-old man with known hypertension presented to the emergency department at a district hospital after an episode of chest pain at rest and left arm weakness. The chest pain was left sided and heavy in nature, radiating into the left arm. He also reported transient weakness and paresthesia in the left arm and a brief loss of consciousness.

**Take-Home Messages**

• Preoperative CT imaging is crucial in patients at risk of aortoiliac occlusive disease to identify critical collateral circulation as unrecognized dependence on the internal mammary or other key vessels can lead to catastrophic limb ischemia.
• Management should be individualized through multidisciplinary evaluation as standard coronary strategies may not account for complex peripheral anatomy or high-risk presentations.

## Past Medical History

The patient was an ex-smoker with a 30 pack-year history, with hypertension and a history of short-distance intermittent claudication at 50 m of exertion. Clinical examination of the lower limbs was negative for hallmarks typical of peripheral artery disease (PAD), such as pulse abnormalities, trophic skin changes, muscle wasting, or ulceration.

## Differential Diagnosis

Electrocardiogram and biochemical findings indicated non–ST-segment elevation myocardial infarction (NSTEMI). Previous cerebrovascular accident was excluded after head computed tomography (CT). A less likely diagnosis was unstable angina or upper limb claudication.

## Investigations

With a positive rise in troponin of more than 20% from baseline measurement and changes on electrocardiogram demonstrating T-wave inversion in leads aVL and I with ST-segment depression in leads V5 and V6, the patient was diagnosed with an NSTEMI and proceeded to coronary angiography.

**Figure 1:**
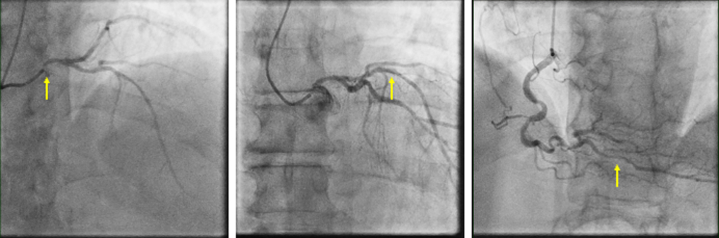
Coronary Angiography Yellow arrows indicate (Left) critical ostial left main stenosis, (Center) mild LAD and LCx atheroma, and (Right) severe distal PDA disease. LAD = left anterior descending artery; LCx = left circumflex artery; PDA = posterior descending artery.

The coronary angiography (Figure 1) revealed critical ostial left main stem disease, with mild atheroma in the left anterior descending artery. Severe disease was noted in the distal posterior descending artery. Mild atherosclerosis was found in the left circumflex and right coronary arteries.

Transthoracic echocardiography revealed severe left ventricular (LV) systolic dysfunction (20%-25%), without LV dilation. After referral to a tertiary unit for consideration of coronary artery bypass grafting (CABG), the patient underwent CT aortogram and cardiac magnetic resonance imaging. The magnetic resonance images suggested that all coronary territories were viable, with a nondilated LV and an ejection fraction of 46%, demonstrating some improvement of contractility after initiation of medical therapy for acute coronary syndrome.

**Figure 2:**
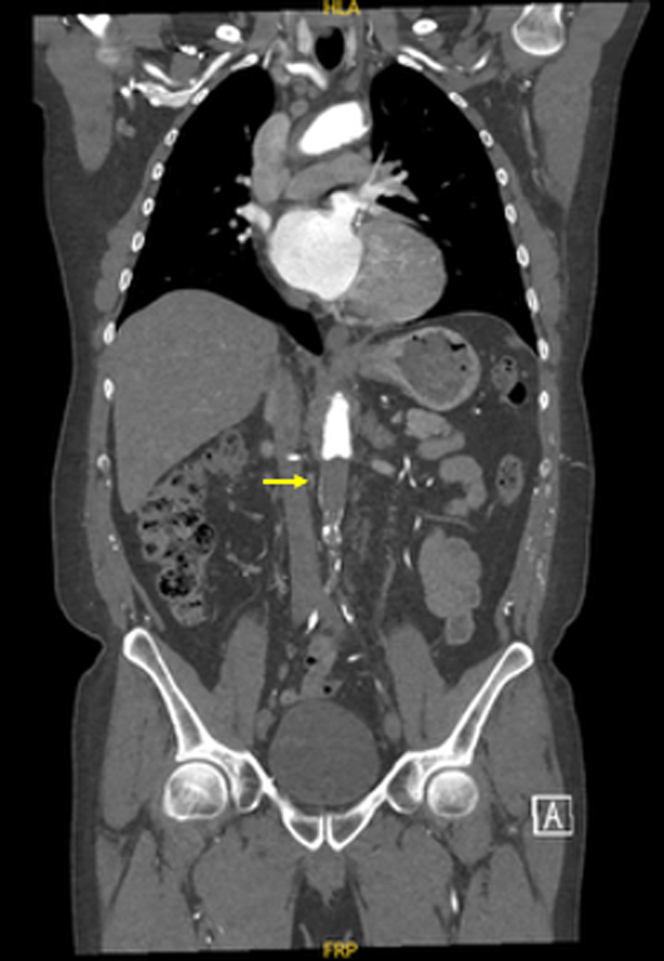
Coronal Image of Computed Tomography Aorta With Arterial Contrast Demonstrating Total Aortic Occlusion

**Figure 3:**
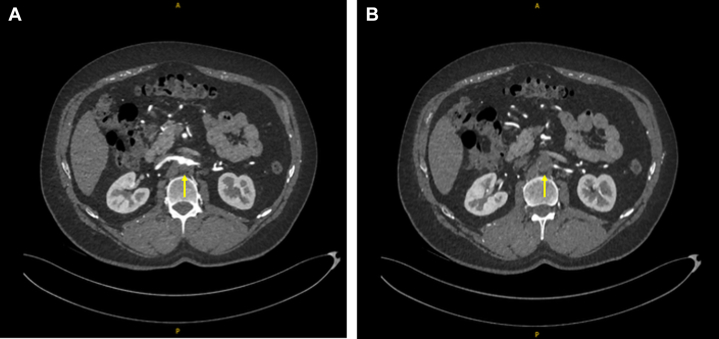
Axial Images From a Computed Tomography Aortogram With Arterial Contrast Demonstrating Complete Infrarenal Aortic Occlusion (Yellow Arrows) (A) Patent proximal right renal artery is shown just above the level of occlusion. (B) The start of the occluded aortic segment is shown.

**Figure 4:**
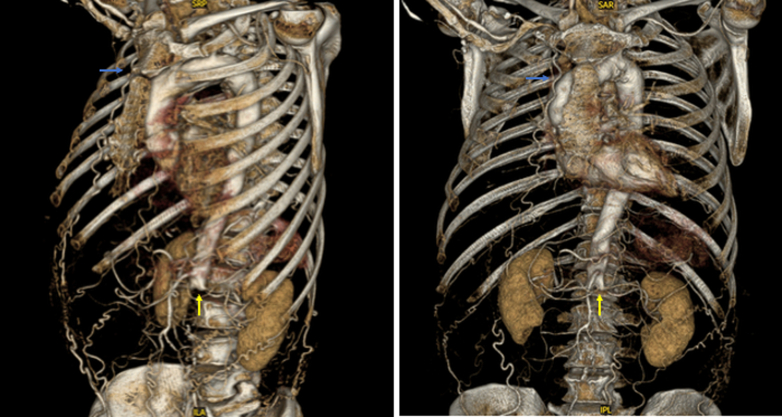
3-Dimensional Reconstruction of Computed Tomography Aortogram With Arterial Contrast Images demonstrate total occlusion of the infrarenal aorta and bilateral iliac arteries (yellow arrows). Contrast opacification is absent distal to the occlusion point just below the renal artery origins. The lumbar and intercostal arteries are faintly visualized, consistent with collateralization. The engorged right internal mammary artery is well visualized (blue arrows), as are its collateralized connections with vessels of the anterior abdominal wall (Winslow pathway).

A CT aortogram, indicated given the patient's history of intermittent claudication, revealed a completely occluded left carotid artery, a completely occluded infrarenal aorta (Figures 2 and 3), and bilateral iliac tracts (type II aortoiliac occlusive disease) (Figure 4). Collateral circulation to the femoral arteries was predominantly via the right subclavian, internal thoracic, and epigastric arteries, and to a lesser extent via the intercostal, subcostal, and lumbar arteries, predominantly via the eponymously named Winslow pathway. The Winslow pathway is a rare but vital route of collateralization between the subclavian and lower limb circulations in cases of severe aortoiliac occlusion. Blood flows from the subclavian artery via the internal mammary arteries to connect into the superior, and then inferior epigastric arteries arising from the external iliac system. This channel allows retrograde perfusion of the pelvis and lower limbs, acting as an extra-anatomical pathway to bypass the occluded aorta. Supporting collateral networks from the lumbar, intercostal (as seen in Figure 4), and subcostal arteries reinforce the system and create the compensatory circulation that maintains distal perfusion.

## Management

At the presenting hospital the patient was treated according to the protocol for acute coronary syndrome and was started on aspirin and a statin. He was then transferred to a cardiac surgery unit for consideration of CABG.

After extensive discussion in a high-risk multidisciplinary meeting involving cardiac surgeons, cardiologists, anesthesiologists, and intensive care physicians, surgery was thought to be too high risk and would likely result in extensive morbidity or mortality. Given the dependency of the patient's lower body circulation upon both mammary arteries, harvest of either mammary artery was deemed to be very high risk. Furthermore, aortic occlusion precluded the use of intra-aortic balloon pump if required; a need deemed more likely than usual given the patient's poor LV function. The patient was repatriated to the referring hospital for staged percutaneous coronary intervention. The left main stem and ostial left anterior descending arteries, then the posterior descending artery, were stented with drug-eluting stents as part of a staged procedure across 2 days.

## Outcome

The patient was followed up in an outpatient setting after 6 weeks. He was free from symptoms and had experienced no adverse cardiac events.

## Discussion

PAD is common in CABG candidates and is an independent predictor of worse surgical outcomes. Extracardiac arteriopathy therefore remains a contributing determination of perioperative risk within commonly used prediction models such as the EuroSCORE II^1^ and Society of Thoracic Surgeons score.^2^ Aortoiliac occlusive disease may be clinically silent owing to well-developed collateral pathways making it challenging to diagnose in certain patient groups without typical claudicative symptoms.

This case is a representation of aortoiliac occlusive disease that was surprisingly well compensated owing to extensive collateralization, allowing the patient to maintain activity levels and quality of life despite symptoms. These alternative pathways develop depending on the level of aortic occlusion, the patency of collateral paths, and the severity of occlusion. Two such dominant pathways have been documented in the literature: systemic-systemic and visceral-systemic, and the much less common Winslow pathway.

A systemic-systemic pathway, with embryological origins in the dorsal aorta, forms via the intercostal, subcostal, and lumbar arteries. These arteries then supply collaterals to the iliolumbar and superior gluteal, with the internal iliac filling in retrograde. A distal abdominal aortic occlusion tends to collateralize in this manner.^3^ A visceral-systemic pathway is more commonly seen in severe cases, as the aortic occlusion extends cephalad toward and above the renal arteries. This pathway connects visceral supply such as the superior mesenteric artery to the inferior mesenteric artery, then via rectal collaterals filling the branches of the internal iliac.^4^ The Winslow pathway, a less common occurrence and as described in the current case, links the internal thoracic artery, superior epigastric artery, inferior epigastric artery, and external iliac artery. This route bypasses the abdominal aorta entirely and can form the predominant blood supply to the lower limbs in cases of infrarenal aortic occlusion.^4^

In the current case, a CT aortogram demonstrated a modified systemic-systemic pattern, with a predominantly right internal thoracic artery serving as key vasculature for lower limb perfusion. Notably however, both mammary arteries were contributing to the collateral network. The collateral network was also involved the costal, epigastric, and lumbar arteries to adequately perfuse the lower limbs despite total occlusion of the infrarenal aorta, with atherosclerotic disease extending into bilateral iliac arteries. Development of such collateral networks is only seen in chronic occlusive states. The left internal mammary artery has long been the gold-standard conduit of choice for patients undergoing CABG, and the right internal mammary artery is an increasingly frequent choice for CABG owing to perceived improved patency rates, lower postoperative cardiac events, and reduced risk of cardiac reoperation of arterial conduits for some patients when compared with saphenous vein grafts.^5^ Many patient factors commonly associated with ischemic heart disease often promote the development of aortoiliac occlusive disease/PAD, and so its presence is common among patients undergoing CABG.^6^^,^^7^ CT angiography is therefore a critical preoperative investigation in patients with suspected or established PAD. Indications include absent femoral pulses, claudication, prior vascular surgery, or extensive aortic calcification on fluoroscopy. Furthermore, this challenging clinical scenario introduced additional perioperative concerns. In the context of such significant PAD, the harvest of venous conduits from the lower limbs would likely post an above-average risk of wound complications such as delayed healing or dehiscence. Complete aortic occlusion would also preclude the use of intra-aortic balloon if necessary, with requirement for such a device being above average in the context of our patient's poor preoperative LV function.

In this patient, the constellation of severe CAD, left main disease, and LV dysfunction would typically warrant CABG. However, the risks of internal mammary artery–dependent collateral disruption, combined with total aortic occlusion foregoing use of an intra-aortic balloon pump and concerns of wound complications relating to venous harvest deemed percutaneous coronary intervention the safer revascularization strategy. Use of the internal thoracic arteries for coronary revascularization in this patient is likely to have compromised lower limb perfusion and may have resulted in acute ischemia.^7^

## Conclusions

Drawing back to our title, this case demonstrates how the mammary arteries, particularly the right internal mammary artery, can function as a lifeline by sustaining perfusion to the lower body in the setting of complete aortic occlusion. Instead of serving their usual role as coronary conduits, in our patient they have undergone adaptive enlargement and collateralization through the Winslow pathway. This resulted in the mammaries becoming the dominant arterial supply to the pelvis and lower limbs as a compensatory mechanism for aortic obstruction. As a result, these commonly used conduits were transformed from a potential graft into an irreplaceable systematic conduit, and the unpredictability of their harvest in these circumstances precluded their use for CABG in similar circumstances. This case highlights how detailed vascular imaging in selected CABG candidates can uncover critical collateral pathways that fundamentally alter management. Multidisciplinary evaluation and individualized planning are essential in the presence of extracardiac arteriopathy.

## Funding Support and Author Disclosures

Dr Layton was supported by the British Heart Foundation, UK (FS/CRTF/24/24703), Heart Research UK, and the van Geest Foundation Heart and Cardiovascular Diseases Research Fund from the University of Leicester, Leicester, UK, 2022. Dr Zakkar was supported by an award from the British Heart Foundation (CH/12/1/29419) to the University of Leicester and Leicester NIHR Biomedical Research Centre, Leicester, UK (NIHR203327). The authors have reported that they have no relationships relevant to the contents of this paper to disclose.

**Visual Summary:** Timeline of the Case

Time	Events
Day 1	A 61-year-old man presented to the emergency department with chest pain and left arm weakness. He was diagnosed with NSTEMI given ECG changes and elevated troponin levels.
Day 2	The patient underwent diagnostic coronary angiography, which demonstrated severe ostial disease in both the left main stem and PDA. TTE demonstrated severe LV impairment, with an ejection fraction of 25%.
Day 3	A multidisciplinary meeting of the heart team recommended referral to the local surgical center for consideration of CABG, in line with guidelines given presence of severe left main stem disease in the context of heart failure.
Day 4	The patient was transferred to the surgical center and, given his history of short-distance claudication, a CT aortogram was performed. This demonstrated total occlusion of the aorta infrarenally, with collateralization via the Winslow pathway. Collateral circulation was largely dependent on the RIMA.
Day 5	Further multidisciplinary team discussion deemed CABG would pose extremely high morbidity and mortality and therefore recommended PCI.
Day 10	The patient underwent successful staged PCI, initially to the left main stem and then to the PDA, using drug-eluting stents. He was then discharged home.

ECG = electrocardiogram; CABG = coronary artery bypass grafting; CT = computed tomography; LV = left ventricular; NSTEMI = non–ST-segment elevation myocardial infarction; PCI = percutaneous coronary intervention; PDA = posterior descending artery; RIMA = right internal mammary artery; TTE = transthoracic echocardiography.

## References

[1] Nashef S.A., Roques F., Sharples L.D. (2012). EuroSCORE II. Eur J Cardiothorac Surg.

[2] Shahian D.M., O'Brien S.M., Filardo G. (2009). The society of thoracic surgeons 2008 cardiac surgery risk models: part 1--coronary artery bypass grafting surgery. Ann Thorac Surg.

[3] Hardman R.L., Lopera J.E., Cardan R.A., Trimmer C.K., Josephs S.C. (2011). Common and rare collateral pathways in aortoiliac occlusive disease: a pictorial essay. AJR Am J Roentgenol.

[4] Prager R.J., Akin J.R., Akin G.C., Binder R.J. (1977). Winslow's pathway: a rare collateral channel in infrarenal aortic occlusion. Am J Roentgenol.

[5] Gaudino M., Lytle B. (2022). Right internal thoracic artery for coronary bypass surgery: did we get it wrong?. Circulation.

[6] Brown J.C., Gerhardt T.E., Kwon E. (2025). StatPearls.

[7] Song P., Fang Z., Wang H. (2020). Global and regional prevalence, burden, and risk factors for carotid atherosclerosis: a systematic review, meta-analysis, and modelling study. Lancet Glob Health.

